# Outpatient minor oral surgery in patients with hemophilia: A case series of 23 patients

**DOI:** 10.4317/jced.55506

**Published:** 2019-04-01

**Authors:** Emilio A. Ramos, Maximiliano Diamante, Diego Caruso, Juan M. Muiño, Alejandra Baques, Ludmila B. Antonelli, Josefina Gutierrez, Marcelo O. Minigutti, Gabriela Guerrero

**Affiliations:** 1 DDS. Maxillofacial Surgery Department Staff, Dr. César Milstein Hospital, Argentina; 2 DDS. Head of the Maxillofacial Department, Dr. César Milstein Hospital, Argentina; 3 MD, MSc. Head of the Clinical Research Department, Dr. César Milstein Hospital, Argentina; 4 DDS. Residents instructor, Maxillofacial Surgery Department, Dr. César Milstein Hospital, Argentina; 5 MD. Hemophilia and Complex Coagulopathies Department Staff, Dr. César Milstein Hospital, Argentina; 6 DDS. Chief Resident, Maxillofacial Surgery Department, Dr. César Milstein Hospital, Argentina; 7 DDS. Resident, Maxillofacial Surgery Department, Dr. César Milstein Hospital, Argentina; 8 MD. Hemophilia and Complex Coagulopathies Department Staff, Dr. César Milstein Hospital, Argentina

## Abstract

Hemophilia is an inherited coagulation disorder characterized by deficiency of the coagulation factor VIII or IX. When the management of hemostasis is inadequate, these patients are at high risk of experiencing intra and postoperative bleedings after tooth extractions. Coagulation factor replacement therapy allows performing most surgical procedures safely, although the factor levels and length of treatment have not been clearly determined. In this study, we present our experience in a retrospective series of 112 ambulatory tooth extractions under local anesthesia in 23 patients with hemophilia using a coagulation factor replacement therapy in combination with tranexamic acid. The results obtained with this protocol were satisfactory and only one episode of mild postoperative bleeding occurred after seven days in a patient who did not have good treatment compliance.

** Key words:**Hemophilia, factor replacement therapy, tooth extractions, postoperative complications.

## Introduction

Hemophilia is an inherited X chromosome-linked bleeding disorder characterized by deficiency of the coagulation factor VIII (FVIII) or IX (FIX). It is estimated that it affects approximately 400,000 subjects worldwide (1). The deficiency of FVIII and FIX is called hemophilia A (HA) and hemophilia B (HB) respectively. Eighty percent have type A (2). Depending on the FVIII or FIX activity, it may be classified in: severe (< 1%), moderate (1-5%) and mild (> 5 and < 40% of normal) (3). The main characteristic of hemophilia is a tendency to bleeding and, consequently, these patients are at risk of experiencing intra and postoperative bleeding episodes after oral surgery procedures. The World Federation of Hemophilia recommends treating the factor deficiency with the specific concentrate, whenever possible, and that the factor levels be returned to normal prior to performing any invasive procedure. In addition to factor replacement therapy, the World Federation of Hemophilia recommends the use of tranexamic acid as an effective means for clot stabilization (2). In this article, we present our experience in a retrospective series of 112 tooth extractions using factor replacement therapy in combination with tranexamic acid. 

## Case Report

Patients over 18 years old with diagnosed HA and HB who underwent tooth extractions at our facility from June 2012 to June 2017 were included. Routine preoperative tests including factor VIII/ IX dosage and screening tests and inhibitor assays were carried out. All the procedures were carried out by the same surgical team on an outpatient basis, in a hospital setting and under local anesthesia. Factor VIII (ADVATE®, Koate®, Kogenate®, Xyntha®) or IX concentrates (Berinin®, Inmunine) were administered approximately thirty minutes before the anesthetic infiltration in order to maintain the factor levels above 60%.

To calculate the factor VIII preoperative dose in patients with hemophilia A, the following formula was used: Dose (IU)= body surface (kg) x level of desired factor VIII increase (IU/dL) x 0.5. In patients with HB, the preoperative factor IX dose was calculated using the following formula: Dose (IU)= body surface (kg) x level of desired factor IX increase (IU/dL) x 0.5. In addition, one gram of tranexamic acid was intravenously administered. Local anesthesia with articaine 4% with epinephrine 1:100,000 (Totalcaína Forte, Laboratorios Bernabó, Buenos Aires, Argentina) was administered. Absorbable 3.0 sutures (Vicryl, Ethicon Inc., USA) were used in all cases. No local hemostatic agents were used. After surgery, subsequent doses of factor VIII or IX were administered for 72 hours calculated on the individual patient’s pharmacokinetics in order to maintain the factor levels above 30% during the postoperative period and 2 grams of tranexamic acid orally every 6/8 hours for 72 hours. In all the cases, antibiotic prophylaxis with 2 grams of amoxicillin (Amixen, Laboratorios Bernabó, Buenos Aires, Argentina), orally, one hour prior to the surgical procedure, was indicated. In those cases of acute infection, antibiotic therapy was maintained until the infection resolved. Analgesic therapy with paracetamol 1 gram (Laboratorios Raffo, Buenos Aires, Argentina), orally, was indicated every eight hours. In cases of moderate to severe pain, tramadol 50 milligrams (Calmador 50 mg, Laboratorios Finadiet, Argentina), orally, was indicated every eight hours. All the patients were discharged prior to the 24 hour period and checkups at the Outpatient Clinics every 24 hours were indicated for three days. They were scheduled for a new checkup seven days later. Indications included: no smoking, avoiding hot food and beverages until sensation in mouth has been recovered, not using mouthwash for 48 hours, applying cold locally and avoiding physical activity until the checkup visit was completed on the seventh day.

The main findings are listed in  Table 1,  Table 1 continue. Two hundred and twelve tooth extractions were carried out in 23 patients. The mean age was 43 years old (18-65 years old range). All the patients were males. Thirty surgical procedures were carried out. Nineteen patients (83%) had HA, three patients (13%) had HB and one patient (4%) had HA and Von Willebrand disease. Eighty-nine percent of the patients with HA and 100% of the patients with HB had severe hemophilia. None of the patients developed inhibitors. In patients with HA, the mean baseline factor concentration was 5.2% (standard deviation = 6.27%) and 4.4% (standard deviation = 2.95%) in patients with HB. Chart one shows the factor levels after concentrate administration. No intraoperative complications occurred. On the seventh day, a postoperative minor bleeding occurred in a patient with severe HB (4%) who did not attend the postoperative checkups for factor administration. Bleeding was controlled by local hemostasis maneuvers using gauzes soaked in tranexamic acid and administering 2,400 IU of FIX every 24 hours for 48 hours.

**Table 1:**
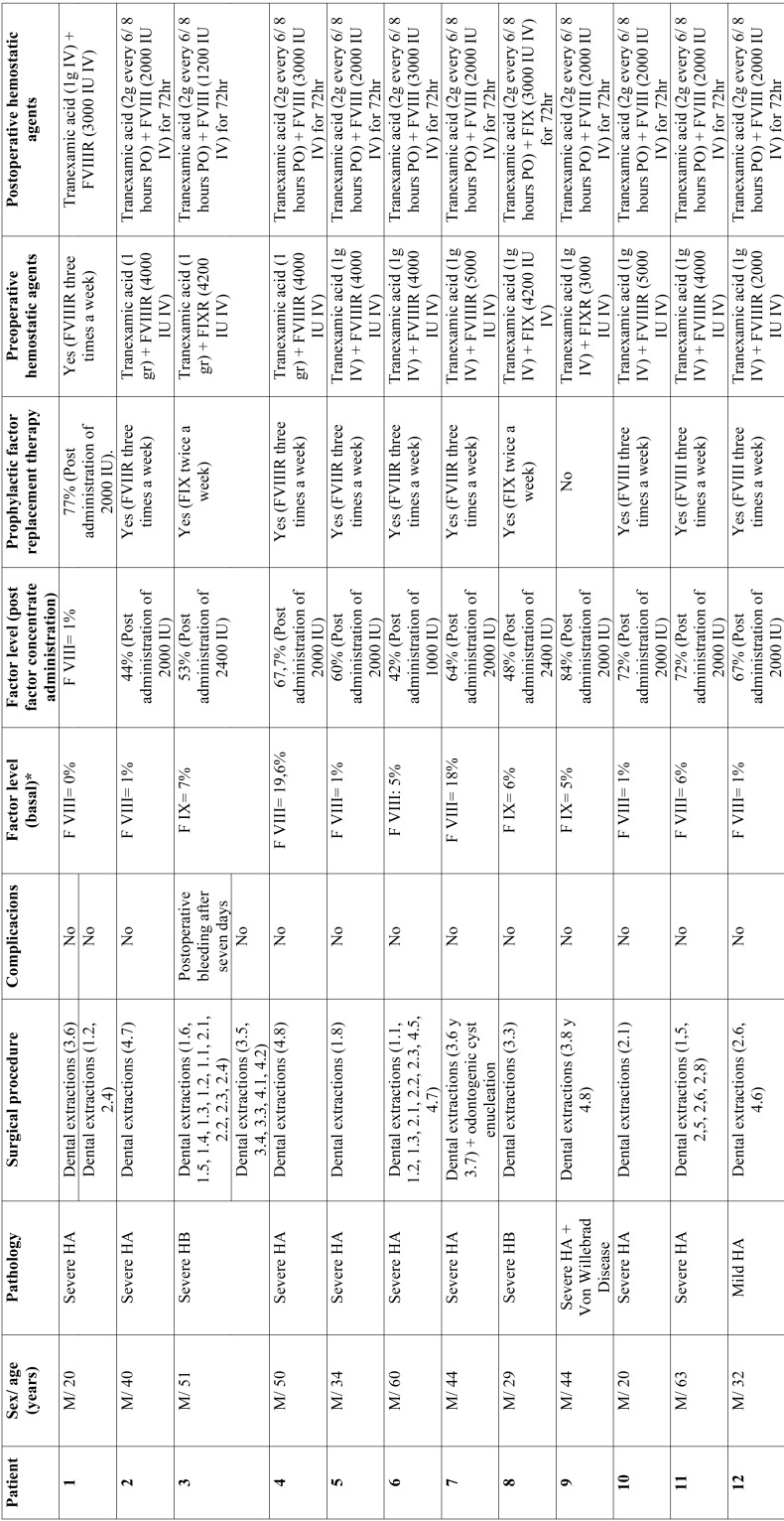
Distribution of patients included in the study, surgical procedure, complications, replacement therapy and hemostatic agents.

**Table 1 continue:**
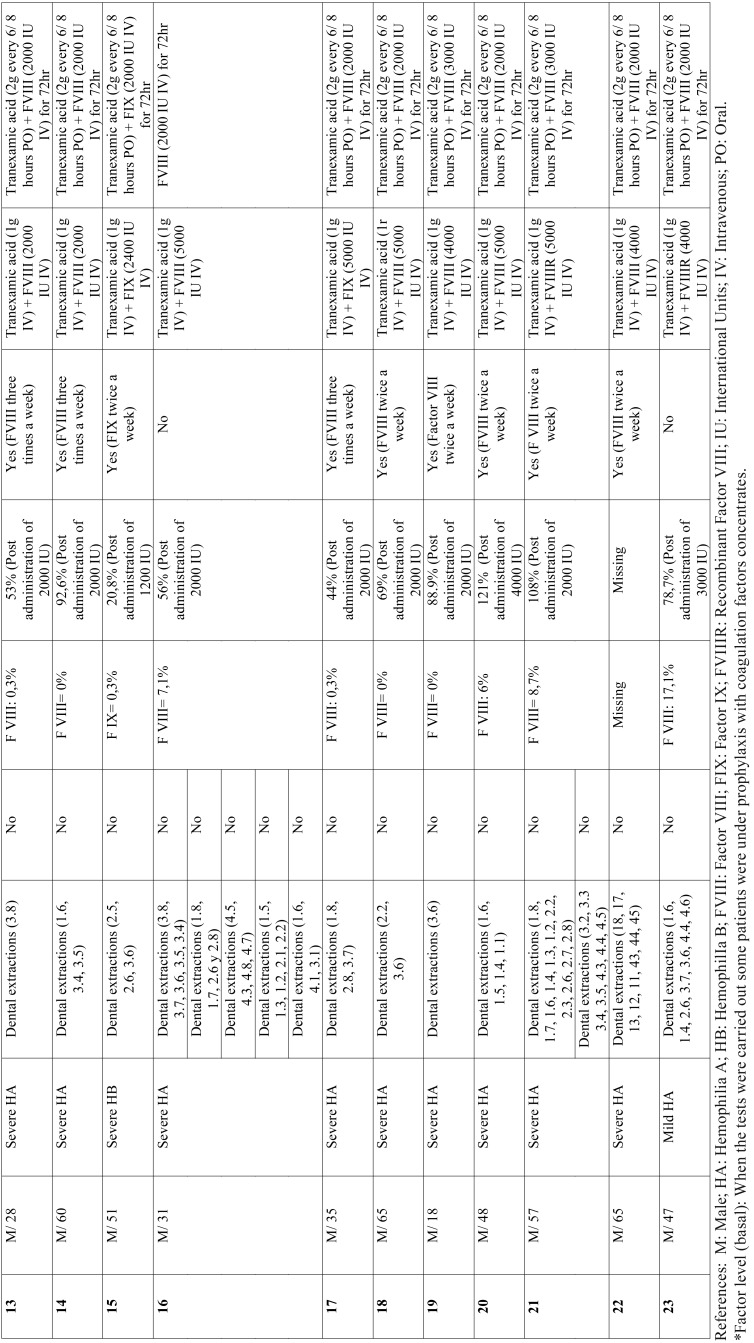
Distribution of patients included in the study, surgical procedure, complications, replacement therapy and hemostatic agents.

## Discussion

Patients with hemophilia are at risk of experiencing intra and postoperative bleeding after oral surgery procedures; consequently, these should be carried out at specialized centers in collaboration with Hematologists with experience in managing this disease (4). The World Federation of Hemophilia recommends treating the factor deficiency with the specific concentrate, whenever possible, and that the factor levels be returned to normal prior to performing any invasive procedure. In this paper, we describe the results obtained in a series of 23 patients who underwent 30 minor oral surgical procedures using a factor replacement therapy in combination with tranexamic acid protocol. A mild case of bleeding occurred in a patient who did not have good compliance with the postoperative instructions. In our series, there were no cases of postoperative infection. The results obtained in our series were similar to those reported by other authors (4-13). At present, the coagulation factor concentrates undergo viral inactivation and consequently the most severe complication related to the hemophilia treatment is developing anti-FVIII and FIX inhibitors (2). The incidence of inhibitor development in patients with severe hemophilia A is of approximately 30% and of 2-4% in patients with severe hemophilia B (13). Inhibitors were not detected in any of the patients included in our series. The mean baseline factor concentration was 5.2% in patients with HA (standard deviation= 6.27%) and 4.4% (standard deviation= 2.95%) in patients with HB. These apparently high values can be explained by the fact that some of these patients were under prophylaxis with coagulation factors when the tests were carried out.

Our protocol includes tranexamic acid administration as coadjuvant agent in combination with factor replacement therapy. In 2015, Watterson et al. published a systematic review on the use of adjuvant therapy with antifibrinolytics in oral surgery procedures in patients with hereditary coagulation disorders. Although the quality of the evidence available is low, there seems to be a benefit in reducing bleeding with no significant increase in adverse effects (14).

Thirty tooth extraction procedures were carried out and one postoperative episode of bleeding occurred. The results obtained in our series by using this protocol were satisfactory, although compliance with treatment and especially with postoperative checkups is something to take into account in order to reduce the risk of complications.
